# Algae

**DOI:** 10.1038/s41598-024-75452-8

**Published:** 2025-01-15

**Authors:** Diego F. Gomez-Casati, Mathieu Pernice, Thierry Tonon

**Affiliations:** 1 Centro de Estudios Fotosintéticos y Bioquímicos (CEFOBI-CONICET), Facultad de Ciencias Bioquímicas y Farmacéuticas, Universidad Nacional de Rosario, Suipacha 531, Rosario, Santa Fe Argentina; 2 Faculty of Science, Climate Change Cluster (C3), University of Technology Sydney, Sydney, NSW 2007 Australia; 3 Department of Biology, Centre for Novel Agricultural Products, University of York, York, YO10 5DD UK

**Keywords:** Metabolism, Ocean sciences

## Abstract

Algae are versatile photosynthetic organisms, with remarkable adaptability and metabolic properties that allow them to live in diverse and extreme habitats, as well as holding great potential for biotechnology. They play fundamental roles in their environments, including primary production, carbon fixation, and engineering their ecosystems. Advances in research on these organisms have been lagging behind bacteria, fungi, plants and animals, but the situation is rapidly evolving thanks to the development of new tools and resources. In this context, the current Collection covers diverse topics including the biology, ecology, conservation and biotechnology of algae.

Algae are a fascinating and diverse polyphyletic group of mostly photosynthetic organisms. These life forms range dramatically in size and complexity, from microscopic unicellular microalgae to giant macroalgae that can grow to the size of trees. Unlike vascular plants, algae do not possess traditional morphological structures such as roots, stems, or leaves. However, they have successfully colonized an astonishing array of environments on Earth, from the depths of the ocean to the surfaces of rocks, and extreme environments like hot springs and arid deserts. Algae play key roles in many ecosystems exemplified by coral reefs in the tropics or dense kelp forests along temperate coastlines, which provide habitat and food for diverse marine life and significantly contribute to coastal biodiversity.

Despite their fundamental importance and increasing interest for biotechnological applications, we still have limited knowledge on different aspects of algae. This collection invited original research to fill knowledge gaps in algae across research areas such as their biology, ecology, conservation, and biotechnology.

One of the most compelling attributes of algae is their ability to absorb large volumes of carbon dioxide (CO_2_) from the atmosphere via photosynthesis. This capability positions them as critical players in primary production and global carbon cycles in marine and freshwater ecosystems. Coral reefs highlight the crucial role of algae and their vulnerability to climate change. *Symbiodiniaceae*, microalgae symbionts living within coral tissues, provide up to 90% of the coral’s carbon through photosynthesis, fuelling reef productivity. During heat stress, the host expels these algae, leading to coral bleaching and energy loss, making reefs highly susceptible to climate change. Matthews et al.^1^ examined how three bacterial partners (*Labrenzia alexandrii, Marinobacter adhaerens, Muricauda aquimarina*) impact the photophysiology of two *Symbiodiniaceae* species. Certain algae-bacteria combinations enhanced photosynthesis and photoprotection, emphasizing bacterial symbionts’ role in supporting coral resilience under warming conditions.

The calcified red macroalga *Lithophyllum byssoides* is a key ecosystem engineer in the mid-littoral zones of the Western Mediterranean Sea. It forms sturdy bioconstructions near sea level that take centuries to develop, serving as markers of historical sea levels. Blanfune et al.^2^ studied these structures in Marseille and Corsica, creating a health index for *L. byssoides* rims. Their findings highlight that sea level rise, driven by climate change, is the primary threat to these vital formations, signalling potential ecosystem decline in affected marine areas.

Light is crucial for algal growth. Summers et al.^3^ found that common Arctic macroalgae (green, red, and brown) could grow new tissues in darkness during the Polar Night, with their photosynthetic apparatus still functioning. They emphasized the value of combining photophysiological data with underwater habitat mapping using remotely operated vehicles equipped with hyperspectral imagers.

Moving from the Arctic to the North Pacific Ocean, Richards Donà et al.^4^ studied groundwater-dependent ecosystems (GDEs) on Hawaii’s Kona coast, where native algae thrive on nutrient-rich submarine groundwater discharge. They found that urban development and groundwater withdrawal favour invasive algae like *Hypnea musciformis* over natives like *Ulva lactuca*, stressing the need for improved land and water management to protect GDEs.

In addition to their primary productivity and vast diversity, algae have recently garnered significant interest as sustainable sources for an array of products. They are increasingly recognized for their biotechnological potential in producing a variety of compounds that are widely used across multiple industries, including food, cosmetics, and pharmaceuticals. In line with this, seaweed farming is considered one of the most sustainable types of aquaculture, and offshore cultivation is being developed for some macroalgae. However, factors like nitrogen availability, wave intensity, and depth must be considered. Zollman et al.^5^ studied how these variables, along with pre-cultivation fertilization, impact the growth and chemical composition of *Ulva* sp. Their predictive model, tested off the Israeli coast, showed that deeper cultivation improved growth due to reduced surface wave impact, and suggested that sufficient nutrients should be provided before offshore cultivation.

Microalgae cultivation is also on the rise. Assobhi et al.^6^ investigated growth and metabolic changes of new isolates of the green microalgae *Chlorococcum* sp. and *Chlamydomonas debaryana* from Morocco under changing salinity or variable nutrient (nitrogen and phosphorus) concentrations. Their results show that these different abiotic conditions had variable impact on the growth and carbon partitioning between carbohydrate and lipid production in the strains investigated. These are important considerations to potentially use *C. debaryana* for bioethanol production and *Chlorococcum* sp. as feedstock for biodiesel.

Pigments from algae, like phycocyanin, chlorophylls, astaxanthin, and fucoxanthin, are used in industries such as food, cosmetics, and health. Fucoxanthin enhances photosynthesis and is expected to see growing demand in nutraceuticals sector thanks to its health benefits including antioxidant properties. However, its levels vary across species and seasons, complicating commercial use. Cunningham et al.^7^ found *Fucus serratus* and *Fucus vesiculosus* had the highest fucoxanthin levels among the four species of brown algae they investigated, but concentrations fluctuated seasonally. Meanwhile, Macdonald-Miller et al.^8^ used mutagenesis to boost fucoxanthin in the diatom *Phaeodactylum tricornutum*, though most mutants reverted to wild-type. Monitoring stability over time is therefore crucial for industrial use. In another study on the biotechnological potential of algae, Li et al.^9^ explored the freshwater microalga *Nannochloropsis limnetica* for bioremediation of dairy waste. Their study highlights its potential for a combination of bioremediating and valorising dairy side streams, resulting in the production of valuable compounds like ß-galactosidase. In the same vein, Gopalakrishnan et al.^10^ considered response surface methodology to assess the potential of co-cultivating symbiotic indigenous wastewater microalgae and bacteria under different physiological conditions to increase microalgal biomass and lipid productivity. This work paved the way for the optimization of cultivation parameters within complex wastewater environments for cost-efficient bioenergy production.

Ricky and Shanthakumar^11^ addressed a growing environmental concern: the presence of pharmaceutical contaminants in aquatic environments. Their study focuses on the antibiotics ciprofloxacin and norfloxacin, which threaten ecosystems and contribute to antibiotic resistance. These authors explored phycoremediation using five algae species: *Chlorella vulgaris, Chlorella pyrenoidosa, Scenedesmus obliquus, Tetradesmus* sp., and *Monoraphidium* sp. They found *S. obliquus* to be the most effective at removing both antibiotics. This research highlights *S. obliquus* as a promising bioremediation tool and opens the door for future studies on safer biotransformation products and potential applications.

Despite their fundamental importance and increasing interest for biotechnological applications, we still have limited knowledge on different aspects of algae biology. The study presented by Liu et al.^12^ allows us to learn more about algal growth and physiology. Using a microfluidic platform and *Chlamydomonas reinhardtii*, they precisely controlled light and nutrient levels, overcoming limitations of traditional growth assays. By varying light and nitrogen, they developed a multiplicative model showing how both factors independently affect growth. This model aids in predicting algae’s response to environmental changes and optimizing bioreactor conditions for maximum biomass production.

Wu et al.^13^ developed PhaeoEpiView, an epigenome browser for the newly assembled genome of the diatom *Phaeodactylum tricornutum*, advancing diatom epigenetics. This tool addresses the gap between genome sequencing and epigenomic annotations, enabling researchers to explore epigenetic modifications like DNA methylation and histone marks alongside genetic data. By integrating these layers, PhaeoEpiView enhances understanding of how diatoms regulate gene expression and respond to environmental signals, accelerating research on diatom adaptation and stress responses, with potential implications for broader epigenetic studies.

Tounosu et al.^14^ explored auxin function in the alga *Klebsormidium nitens*, a streptophyte lacking the typical TIR1/AFB auxin receptor found in land plants. Despite this, auxins still influence gene expression in *K. nitens*, suggesting alternative signalling mechanisms. The study identified motifs in auxin-inducible gene promoters and focused on the transcription factor KnRAV, laying the groundwork for further research into this unique auxin response system. Future studies will need to clarify KnRAV’s role and identify other components of this alternative pathway to better understand auxin regulation in algae.

Algae represent a diverse group of photosynthetic organisms that have adapted to thrive in a wide range of environments. Thanks to increasing interest in algae and progress in technology, research on algae is expanding rapidly. With the emergence of an “algae bioeconomy”, there is also a growing number of stakeholders interested in algae as our understanding of these organisms unfolds. However, boundaries still need to be pushed to better understand the biology of algae and to harness their potential in delivering effective solutions to the challenges faced by our rapidly evolving world and to benefit society. Studies like those presented in this Collection contribute valuable insights into algae’s biology, ecology, and biotechnological applications, paving the way for informed conservation and innovation efforts in the future.
